# Vector competence of *Aedes vexans* (Meigen), *Culex poicilipes* (Theobald) and *Cx. quinquefasciatus* Say from Senegal for West and East African lineages of Rift Valley fever virus

**DOI:** 10.1186/s13071-016-1383-y

**Published:** 2016-02-20

**Authors:** El Hadji Ndiaye, Gamou Fall, Alioune Gaye, Ndeye Sakha Bob, Cheikh Talla, Cheikh Tidiane Diagne, Diawo Diallo, Yamar BA, Ibrahima Dia, Alain Kohl, Amadou Alpha Sall, Mawlouth Diallo

**Affiliations:** Unité d’Entomologie Médicale, Institut Pasteur de Dakar, 36 Avenue Pasteur, BP 220 Dakar, Senegal; Université Cheikh Anta Diop de Dakar, Département de Biologie Animale, Faculté des Sciences et Techniques, Dakar, Senegal; Institut Pasteur de Dakar, Unité des Arbovirus et Virus de Fièvres hémorragiques, Dakar, Senegal; MRC-University of Glasgow Centre for Virus Research, Glasgow, G61 1QH Scotland UK

**Keywords:** Mosquito, Oral infection, Vector competence, Viral genetic diversity, Rift Valley fever virus, Senegal

## Abstract

**Background:**

Rift Valley fever virus (RVFV; *Phlebovirus*, *Bunyaviridae*) is a mosquito–borne, zoonotic pathogen. In Senegal, RVFV was first isolated in 1974 from *Aedes dalzieli* (Theobald) and thereafter from *Ae. fowleri* (de Charmoy)*, Ae. ochraceus* Theobald*, Ae. vexans* (Meigen)*, Culex poicilipes* (Theobald)*, Mansonia africana* (Theobald) and *Ma. uniformis* (Theobald). However, the vector competence of these local species has never been demonstrated making hypothetical the transmission cycle proposed for West Africa based on serological data and mosquito isolates.

**Methods:**

*Aedes vexans* and *Cx. poicilipes,* two common mosquito species most frequently associated with RVFV in Senegal, and *Cx. quinquefasciatus*, the most common domestic species, were assessed after oral feeding with three RVFV strains of the West and East/central African lineages. Fully engorged mosquitoes (420 *Ae. vexans*, 563 *Cx. quinquefasciatus* and 380 *Cx. poicilipes*) were maintained at 27 ± 1 °C and 70–80 % relative humidity. The saliva, legs/wings and bodies were tested individually for the RVFV genome using real-time RT-PCR at 5, 10, 15 and 20 days post exposure (dpe) to estimate the infection, dissemination, and transmission rates. Genotypic characterisation of the 3 strains used were performed to identify factors underlying the different patterns of transmission.

**Results:**

The infection rates varied between 30.0–85.0 % for *Ae. vexans*, 3.3–27 % for *Cx. quinquefasciatus* and 8.3–46.7 % for *Cx. poicilipes*, and the dissemination rates varied between 10.5–37 % for *Ae. vexans*, 9.5–28.6 % for *Cx. quinquefasciatus* and 3.0–40.9 % for *Cx. poicilipes*. However only the East African lineage was transmitted, with transmission rates varying between 13.3–33.3 % in *Ae. vexan*s, 50 % in *Cx. quinquefasciatus* and 11.1 % in *Cx. poicilipes. Culex* mosquitoes were less susceptible to infection than *Ae. vexans*. Compared to other strains, amino acid variation in the NSs M segment proteins of the East African RVFV lineage human-derived strain SH172805, might explain the differences in transmission potential.

**Conclusion:**

Our findings revealed that all the species tested were competent for RVFV with a significant more important role of *Ae. vexans* compared to *Culex* species and a highest potential of the East African lineage to be transmitted.

**Electronic supplementary material:**

The online version of this article (doi:10.1186/s13071-016-1383-y) contains supplementary material, which is available to authorized users.

## Background

Rift Valley fever virus (RVFV) is an emerging mosquito-borne, zoonotic pathogen in the genus *Phlebovirus* of the family *Bunyaviridae*. The virus, first isolated in 1930 in Kenya [1], is transmitted primarily through the bites of infected mosquitoes [2–4]. The genome of the virus consists of three negative-stranded RNA segments called large (L), medium (M), and small (S). Other modes of transmission include direct contact with the blood, organs, foetus, tissues or excretions of infected animals through exposure to aerosols. Rift Valley fever (RVF) in animals is characterised by high rates of abortion in pregnant females and deaths of young ruminants. The vast majority of human infections are asymptomatic, but symptomatic infections can lead to severe haemorrhages, meningoencephalitis, retinopathy and in some cases death [5–8]. The disease has cumulatively caused hundreds of thousands of human infections and the deaths of more than 2000 humans and millions of domestic animals in Kenya [9]. To date, no specific treatment against RVF is available. For humans, the available vaccine is restricted to the use for at-risk personnel only, as multiple inoculations are required to achieve protective immunity [10]. Several veterinary vaccine candidates were proposed (MP12, Clone 13, and the Smithburn neurotropic strain), but adverse effects were observed after vaccination [11–13]. Another candidate vaccine (R566) is still currently under investigation [14]. Recently, the MP-12 virus containing a deletion in the NSs gene proved promising as it prevented lethal disease when administered to hamsters [15]. Nonetheless, this new generation of genetically modified vaccines are not yet approved for use in humans or animals.

The recent expansion of RVFV from Africa into Saudi Arabia and Yemen in 2000, and the outbreaks recorded in Africa, particularly those in Mauritania in 2003, 2010 and 2012 [16–19], in Senegal in 2012 and 2013 [20], in Kenya [21, 22], and in Mayotte in 2008 [23], reflect the ongoing emerging potential of the virus. The introduction and spread of the virus into new areas were mainly linked to the migration of infected animals [24], but the dispersal of infected vectors could not be ruled out. As no specific treatment against the RVFV is available, prevention is key, and control of epidemics centred on vector control and the vaccination of cattle and populations at risk (i.e., veterinarians, slaughterhouse staff and medical surveillance personnel, among others).

In Senegal, RVFV was first isolated in 1974 from the mosquito *Ae. dalzieli* collected in Kedougou during a Yellow Fever (YF) surveillance programme. Following the first extensive RVFV outbreak in Mauritania in 1987, the active surveillance programme implemented resulted in the detection of several animal cases and also the isolation of the virus from several mosquito species, including *Ae. fowleri, Ae. ochraceus, Ae. vexans, Cx. poicilipes, Ma.. uniformis* and *Ma. africana* [25–27].

Based on isolates from mosquitoes and the sero-epidemiological data gathered from animals, a transmission cycle similar to that of East Africa was proposed for West Africa. However, this transmission cycle is still hypothetical, because specific entomological parameters such as vector competence of the local species of mosquito for RVFV remain to be determined [28]. Furthermore, although entomological surveillance provided essential information on circulation of the virus and ecology of the vectors, this approach did not determine the effect of RVFV amplification on human populations. Human cases of RVFV are still rare in Senegal [29]. The data generated during a trans-sectional study and following an epizootic event revealed a low seroprevalence of IgG in human populations that ranged from 14.02–22.3 % in 1989 in Yonoféré [30] and 6.12 % in Barkédji in 1993 [29]. The seroprevalence was from 5–26 % amongst children born after the 1987 epidemic, and 25.3 % amongst adults [28, 29]. A low seroprevalence of IgG against RVFV during an investigation of an epidemic was also recorded in Diawara, northern Senegal (5.2 %), and in the south of the country (≤3.1 %) in Kedougou, Tambacounda and Casamance [31–33]. Several factors could explain the difference in infection rates between animals and humans, including the efficiency of transmission by the vector or ecological parameters of the vector.

The purpose of the present study was to investigate the vector competence of *Ae. vexans* and *Cx. poicilipes,* the two most common mosquito species in the Barkédji area and the species most frequently associated with the RVFV in Senegal, and that of *Cx. quinquefasciatus*, the most common domestic species, for different strains and lineages of the RVFV.

## Methods

### Mosquito species

The mosquito species *Ae. vexans, Cx. poicilipes* and *Cx. quinquefasciatus* were collected in Barkédji (15°17′N, 14°53′W). *Aedes vexans* and *Cx. quinquefasciatus* were chosen as target organisms due to their widespread Afrotropical distribution and potential involvement in the transmission cycle of RVFV in Senegal and neighbouring countries [26, 28]. *Aedes vexans* is regularly found naturally infected with RVFV in West Africa [25, 27, 34], has a worldwide distribution and is a biting nuisance pest also in Europe and America. The wide distribution of *Ae. vexans* is a major concern because of the potential for RVFV to invade new geographic areas, as occurred during the epidemic / epizootic in Saudi Arabia [35, 36]. In West Africa, the abundance and the biology of *Ae. vexans*, including the close interactions with vertebrate hosts, highlight the potentially important role that this species may play in the transmission of RVFV.

*Culex quinquefasciatus* was selected because this species is the member of the *Cx. pipiens* complex best adapted to the tropical and sub-tropical regions. *Culex pipiens* was implicated in the transmission of RVFV during an outbreak in Egypt and on the Arabian Peninsula [36, 37]. In tropical regions, *Cx. quinquefasciatus* is ubiquitous, colonizing domestic environments year-round because of its association with artificial breeding sites. This species is abundant, anthropophagic and a competent vector for arboviruses in some geographic locations, making it a good candidate for the study of RVFV transmission in urban settings.

*Culex poicilipes* was targeted in this study as it is considered as one of the main vectors of RVFV in West Africa due to its abundance, bionomics and the number of RVFV strains isolated from this species in Senegal and Mauritania [26, 38]. In our previous studies, the dominance in abundance regularly switched between *Cx. poicilipes* and *Ae. vexans* in Barkédji [39, 40]. Feeding preference studies indicated that *Cx. poicilipes* was mainly attracted to bovines and sheep, adding further indication as to its vectorial potential [41].

### Virus strains and preparation of the stocks

The three RVFV strains used in this study were isolated from goats (AnD133719), mosquitoes (ArD141967) (both West African lineages), and humans (SH172805, East/central African lineage) in Mauritania in the years 1998, 2000, and 2003, respectively (Table 1). The viral stock of each strain used to infect mosquitoes was prepared from the brains of suckling mice inoculated intracerebrally with 20 μl of the virus. Brains were triturated in Leibovitz-15 (L-15) medium (GibcoBRL, Grand Island, NY, USA) containing penicillin and streptomycin (Sigma, GmBh, Germany) and 10 % FBS (Gibco BRL, Grand Island, NY, USA). After centrifugation, the suspension was aliquoted, and stored at −80 °C.

**Table 1:** RVFV strains used in this study, and titre of virus in the infectious blood meal after exposure

Virus strain	Host Origin	Geographic Origin	Year of isolation	Passage history	Lineage	Virus titer post exposure (PFU/ml)		
						*Ae. vexans*	*Cx. quinquefasciatus*	*Cx. poicilipes*
SHM172805	Human	Mauritania	2003	3	East/Central Africa	4.5 × 10^6^	1.5 × 10^6^	9.7 × 10^8^
ArD141967	*Cx. poicilipes*	Mauritania	2000	4	West Africa	5.5 × 10^6^	5.5 × 10^8^	1.1 × 10^7^
AnD133719	Goat	Mauritania	1998	7	West Africa	9.5 × 10^6^	1.7 × 10^6^	3 × 10^6^

### Experimental infection procedure

Three to five day-old F_1_ generation female mosquitoes were starved for 48 h and then exposed for 1 h to an infectious blood meal, using the previously described artificial feeding method using a mouse skin membrane [42]. The infectious blood meal contained at equal volume, washed rabbit erythrocytes, foetal bovine serum (FBS) and the viral suspension of one of the three natural RVFV strains described above. As a phagostimulant, adenosine triphosphate (ATP) was added to a final concentration of 0.005 M. For each infection experiment, a sample of the virus-blood suspension was collected at the end of the mosquito feeding for virus titration. At the end of feeding, the mosquitoes were cold anaesthetised, and fully engorged specimens were selected and subsequently maintained in an incubator at 27 °C, a relative humidity of 70–80 % and 10 % sucrose for food. Between 25 and 40 individuals were randomly selected for each batch at 5 days post exposure (dpe), 25 to 50 at 10 dpe, 36 to 100 at 15 dpe, and 6 to 66 at 20 dpe (Fig. 3). Mosquitoes were again cold anaesthetised, and the legs and wings removed and put together in a tube. Each of these mosquitoes was then forced to salivate individually into a capillary tube containing FBS. After 15–20 min of salivation, the mosquitoes were removed, and the FBS-saliva mix was added into a vial containing 500 μl of L-15 medium.

### Virus detection in mosquitoes

The mosquito bodies, legs/wings and collected saliva were stored separately at −80 °C until virus detection was attempted by real-time RT-PCR. All mosquito bodies as well as the legs/wings of infected bodies and saliva of infected legs/wings were tested for presence of virus. The samples were homogenised in 500 μl of L-15 cell culture medium containing 20 % FBS and were then centrifuged for 20 min at 10,000 rpm at 4 °C. For real-time PCR, 100 μl of supernatant was used for RNA extraction using the QIAamp Viral RNA Extraction Kit (QIAgen, Heiden, Germany), according to the manufacturer’s protocol. The RNA was amplified using ABI Prism 7000 SDS Real-Time apparatus (Applied BioSystems, Foster City, CA, USA) with the QuantiTect kit (QIAgen).

The RT-PCR was performed in a 25 μl reaction volume containing 5 μl of extracted RNA (Triplicate), 2x QuantiTect Probe, RT-Master Mix, 10 μM of each primer and 200 nM of the probe. The specific primers and probe sequences for RVFV used were first described in Weidman et al. [43]. The thermal profile was as follows: a single cycle of reverse transcription for 10 min at 50 °C, 15 min at 95 °C for reverse transcriptase inactivation and DNA polymerase activation, and then 40 amplification cycles of 15 s at 95 °C and of 1 min at 60 °C (annealing-extension step). Fluorescence was analysed at the end of the amplifications.

### Viral RNA extraction, RT-PCR and sequencing

The RNA were extracted from the three viral stocks (AnD133719, SHM172805 and ArD141967) and were used as templates for RT-PCR. Specific primers (Table 2), the M-MLV system (Invitrogen, Carlsbad, CA, USA), and the Go-Taq PCR Kit (Promega, Madison, WI, USA) were used for cDNA synthesis and amplification, according to the manufacturer’s instructions. All primers used to amplify the S and M segments were designed according to RVFV sequences available in GenBank. Accession numbers of all RVFV sequences used to design the primers are presented in Additional file 1: Table S1. The PCR products of the expected sizes were purified directly from the agarose gel using a QIAgen Gel extraction kit and sequenced by Cogenics (Beckman Coulter Genomics, Essex, United Kingdom). Sequencing was performed in both directions, using the original reverse and forward primers as for the amplification.

**Table 2:** List of primers used

Nom	Segment	Position	Sequence	Tm
NSng	S	31–48	TATCATGGATTACTTTCC	48
NSca	S	841–824	CCTTAACCTCTAATCAAC	50
M1F	M	3–22	ACAAAGACCGGTGCAACTTC	53.9
M1R	M	1120–1140	CCAYGCAAAGGGTATGCAAT	53.2
M2F	M	1035–1054	TGAGGACTCTGAATTRCACCT	48.7
M2R	M	2395–2415	TCCAGAGAGTTGAGCCTTGC	53.3
MRV1a	M	3050–3068	CAAATGACTACCAGTCAGC	44.6
MRV2g	M	2262–2292	GGTGGAAGGACTCTGCGA	52.5
M3F	M	2979–2998	CAGTCCTCAGTGAGCYCATA	46.1
M3R	M	3763–3782	TCTCGGTTCTGGRGTGTGAA	52.5

### Data analysis

Detection of RVFV in the mosquito bodies without the subsequent detection of the infection in the legs/wings was considered a non- disseminated infection, which was limited to the midgut. Conversely, detection of the virus in both the mosquito legs/wings and saliva indicated a disseminated infection from the midgut into the mosquito haemocoel and the potential for transmission, respectively. The infection rate (number of infected mosquito bodies per 100 mosquitoes tested), the dissemination rate (number of mosquitoes with infected legs/wing per 100 mosquitoes infected) and the transmission potential (number of mosquitoes with infected saliva per 100 mosquitoes with infected legs/wings) were calculated and compared for each mosquito species, according to dpe and the viral strains. Fisher’s exact tests were performed to compare the rates of infection, dissemination and transmission using the R statistical software package (R Foundation for Statistical Computing, Vienna, Austria). Differences were considered statistically significant at *P* < 0.05.

Nucleotides sequences from Beckman Coulters were analysed and assembled with GeneStudio, version 2.2.0.0 (http://genestudio.com/). The amino acid sequences were then aligned using MEGA 5.05 software [44] to identify variable motifs between the human (SHM172805) and other strains that could explain the different patterns in the mosquito species tested.

## Results

**Fig. 1:**
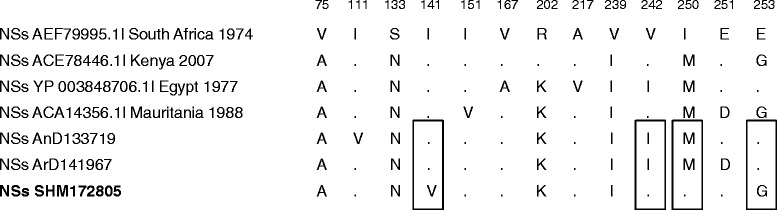
Alignment of S segment. NSs proteins were aligned and variable amino acids were shown with their positions refer to the genome sequence of South Africa 1974 (Accession number: AEF79995.1). Human strain SHM172805 (East/Central African lineage) is in bold. Dots: conserved amino acids. Rectangles: variables amino acids in the human strain sequence compared to ArD141967 and AnD133719 (West African lineage)

**Fig. 2:**
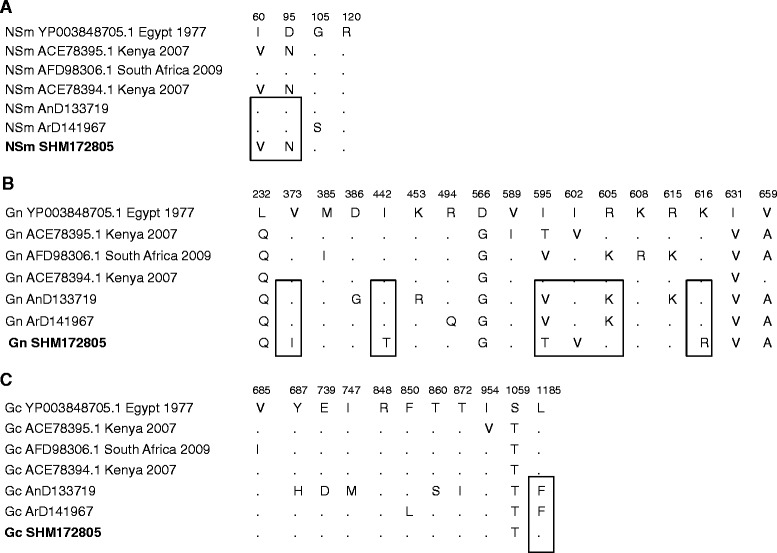
Alignment of M segment. M proteins were aligned and variable amino acids in NSm, Gn and Gc proteins were shown with their positions refer to the genome sequence of Egypt 1977 (Accession number: YP003848705.1). Human strain SHM172805 is in bold. Dots: conserved amino acids. Rectangles: variables amino acids in the human strain sequence compared to ArD141967 and AnD133719

The sequences of the S and M segments of the coding region obtained for the three strains of virus were aligned using MEGA. Based on these alignments, variation in amino acids were observed in the NSs, NSm, Gc and Gn proteins of the human strain (SHM172805) compared to the two other strains (Figs. 1 and 2). This variability occurred primarily among amino acids of the same type (I/V/M and R/K) but also between amino acids of different types, acid/polar, polar/apolar, and aliphatic/aromatic (D/N, V/T, L/F respectively). The N-linked glycosylation sites of the envelope proteins were all conserved.

**Fig. 3:**
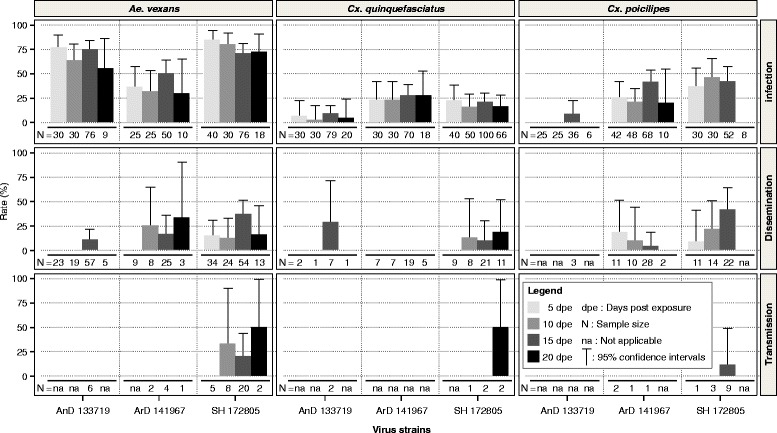
RVFV infection, dissemination and transmission rates throughout incubation periods as indicated for *Ae. vexans, Cx. quinquefasciatus* and *Cx. poicilipes* that were orally exposed to infectious blood meal containing 10^6^-10^8^ PFU/ml virus suspension with RVFV strains SH72805, ArD14196 or AnD133719. N: number of specimens tested. dpe: day post exposure

A total of 420 *Ae. vexans*, 563 *Cx. quinquefasciatus* and 380 *Cx. poicilipes* were tested for RVFV competence. All three virus strains (with virus titers ranging from 1.5× 10^6^ pfu/ml to 9.7 × 10^8^ pfu/ml) infected the three species of mosquito and were disseminated, but only the East African lineage RVFV was capable of being transmitted by all three species. Infection rates varied between 30 and 85 % for *Ae. vexans*, 3.3–27 % for *Cx. quinquefasciatus* and 8.3–46.7 % for *Cx. poicilipes*. For each mosquito-virus combination, the infection rates were comparable at each dpe, as well between the different dpe. However, significant variation was detected when comparing the infection rates by the different virus strains at different dpe (Fig. 3 and Additional file 2: Table S2).

The infection rates of *Ae. vexans* with the ArD141967 strain were significantly lower than those with the SH172805 strain (*P* ≤ 0.04). The same trend was observed between the ArD141967 and the AnD133719 strains (*P* ≤ 0.03), except at 20 dpe (*P* = 0.3). However, no significant difference was recorded between the infection rates of AnD133719 and SH172805 (*P* ≥ 0.25). With *Cx. quinquefasciatus*, the infection rates were similar between SH172805 and ArD141967 strains. AnD133719 was the only strain that exhibited infection rates significantly lower than those of the SH172805 strain (*P* = 0.03) and the ArD141967 strain (*P* = 0.004).

For *Cx. poicilipes*, no significant differences were observed irrespective of dpe (*P* > 0.05), except at 10 and 15 dpe with the ArD141967 strain (*P* = 0.021). Comparison among the viral strains revealed that at 10 dpe, the SH172805 strain was more infectious than the ArD141967 strain (*P* = 0.016), and at 15 dpe, the infection rate of the AnD133719 strain was significantly lower than both the ArD141967 (*P* = 0.00049) and SH172805 strain (*P* = 0.0002).

Based on a pairwise analysis of infection rates between the species, *Ae. vexans* was generally more susceptible to infection than *Cx. quinquefasciatus.* The unique exception was the infection obtained with the ArD141967 strain at 15 dpe, when the infection rate of *Ae. vexans* was significantly higher than that of *Cx. quinquefasciatus* (*P* = 0.001). Indeed using the same strain of virus, a comparable rate of infection was found for *Ae. vexans* and *Cx. quinquefasciatus* at 5, 10, and 20 dpe (*P* ≥ 0.3).

Independent of the virus strain used, dissemination rates were relatively low for the three species of mosquitoes ranging from 10.5–37 % in *Ae. vexans,* from 9.5–28.6 % in *Cx. quinquefasciatus* and from 3–40.9 % in *Cx. poicilipes*. Dissemination was recorded relatively quickly in *Ae. vexans* at 5 dpe for the SH172805 strain but was delayed to 10, and 15 dpe, for the ArD141967 and AnD133719 strains, respectively. With the exception of the significant difference observed at 15 dpe between the AnD133719 and SH172805 strains (*P* = 0.001), the dissemination rates were comparable for all virus strains and incubation periods (*P* ≥ 0.07). In *Cx. quinquefasciatus*, the dissemination rates remain similar irrespective of the dpe (*P* ≥ 0.1). This species did not disseminate the ArD141967 strain, despite the high viral titres of 5.510^8^ PFU/ml detected in the blood meal post-feeding. The observation was similar for *Cx. poicilipes*, which did not disseminate the animal strain (AnD133719).

For all mosquito species tested, only the SH172805 strain was transmitted (i.e. present in saliva) from 10 dpe and thereafter for *Ae. vexans*, 15 dpe for *Cx. poicilipes* and only after 20 dpe for *Cx. quinquefasciatus*. The transmission rates ranged from 13.–33.3 % for *Ae. vexans* and was 11.1 % for *Cx. poicilipes* and 50 % for *Cx. quinquefasciatus*, but the differences between the three species and the dpe were not significant (*P* ≥ 0.05).

## Discussion

Our study showed that the three mosquito species were susceptible to infection by the different RVFV strains with *Ae. vexans* mosquitoes being more susceptible than *Cx. poicilipes* or *Cx. quinquefasciatus*. The highest infection rates were obtained with *Ae. vexans*, which was also the only species that disseminated all the virus strains and transmitted the SH172805 strains at different dpe. Despite a lower viral titre (≈ 10^6^ PFU/ml) in the blood meal post-feeding, the rates of infection, dissemination and transmission rates obtained were as high as those obtained in US populations of *Ae. vexans* that were exposed to a viraemic animal that exceeded or was equal to 10^8.5^ PFU/ml [45]. The rates of infection, dissemination and transmission were 47.9–95.4 %, 10.3–60.8 % and 25–100 %, respectively. However, our results differed from those of other studies [46] that reported absence of competence after exposure to viral titres that ranged from 10^7.9^–10^9^ PFU/ml and led to an infection rate of only 15 % without dissemination or transmission. Geographical diversity in the competence of *Ae. vexans* has already been observed in populations from different localities in the USA. Populations from Louisiana and Florida exhibited 27 % transmission rates, whereas the rate of transmission in colonies from Colorado and California was only 1 % [47]. Indeed, the *Ae. vexans* complex includes three subspecies: *Ae. vexans vexans* (Meigen, 1830) found in Europe, *Ae. vexans nipponii* (Theobald, 1907) from eastern Asia, and *Ae. vexans arabiensis* (Patton, 1905) is the only representative in Africa. Because this study focused on only one strain of each mosquito species and vector competence of mosquito populations can vary spatially, our results may not apply to population from other regions of West Africa. Further studies are needed to evaluate the vector competence of other populations of these species for RVFV.

The low infection rates among the two *Culex* species observed in our study are consistent with previous results of *Cx. quinquefasciatus* from South Africa, Australia and the south-eastern United States [45, 47–50], with a maximum infection rate of 26 %, without dissemination and transmission. The low transmission rate (11.1 %) found here for *Cx. poicilipes* is similar to the results of a previous South African study at 15 dpe [51]. This transmission rate could become higher with longer incubation periods, as it was suggested that the South African population of *Cx. poicilipes* reached 80 % transmission after 30 days of extrinsic incubation.

Only the RVFV strain belonging to the East African lineage (SH172805) was transmitted. These observed variations were not caused by differences in viral titres because no significant correlation was observed between viral titres and infection rates. Contrary to our findings, Turell et al. [47] found that mosquito infection and dissemination rates were higher when mosquitoes were exposed to higher viral doses. This discordance could be explained by differences in mosquito strains and the experimental protocol.

The genetic variability of the virus strains used in our experiment could explain the differences in patterns of infection and transmission. The results of the sequence analysis of a portion of the genome at the L, M and S segments provided some support for this hypothesis. Indeed, in addition to classification into the East African lineage, the alignment of partial segments showed a number of differences between the M and S segments of the SH172805 strain to those of the other West African strains. Amino acid changes were detected in the NSs, NSm, Gc and Gn proteins of the human East African strain compared with those of the West African strains. Notably, the M segment proteins are essential and necessary for virus spread. Indeed the Gn and Gc proteins are implicated in the RVFV entry during infection, although the cell receptor remains unknown [52, 53]. The M segment proteins also play important roles in the assembly of the Golgi and the budding of viral particles [54]. It has been shown that the NSm protein has an anti-apoptotic function and a negative effect on virus development in the cell [55–57]. Additionally, NSm has been described to play a functional role in influencing vector competence for RVFV at the level of the midgut barrier [58]. The NSs protein acts on the antiviral response of mammalian cells by direct or indirect inhibition of transcription factor [59, 60]. Alone or combined, these two proteins (NSs and NSm) affect vector competence of some species by reducing the rate of infection or the potential to transmit the virus [61]. Therefore, the single amino acid mutations observed in all these proteins could influence the viral replication and lead to different patterns of transmission. A single amino acid mutation can indeed enhance transmission in a mosquito vector. In chikungunya virus, a single mutation (A226V) in the E1 glycoprotein enhanced viral transmission by *Ae. albopictus* [62, 63]. Further studies are required to assess the effects of these amino acid differences between RVFV strains. Studies with reassortant viruses from these strains or genetically engineered viruses could pinpoint what segment exactly carries determinants for the competence phenotype to further inform risk analysis. The lower susceptibility of *Cx. quinquefasciatus*, which is the most abundant domestic vector and a highly anthropophilic species may explain why human cases of RVFV are still rare in Senegal. Only the strain belonging to the East African lineage (SH172805) was transmitted, suggesting that this lineage is more infectious for mosquito vectors than the West African lineage in agreement with the fact than more RVFV outbreaks were observed in East Africa compared to West Africa.

Considering that *Ae. vexans, Cx. poicilipes* and *Cx. quinquefasciatus* exhibited a minimum EIP of 10, 15 and 20 days, respectively and given their estimated survival rates obtained from previous studies [64, 65], the infective life expectancy was estimated at between (0.91)^10^ to (0.96)^10^ for *Ae. vexans,* between (0.70)^15^ to (0.79)^15^ for *Cx. poicilipes,* and between (0.871)^20^ and (0.883)^20^ for *Cx. quinquefasciatus*. This means that 38.9–66.4 % of *Ae. vexans,* but only 0.5–2.9 % of *Cx. poicilipes* and 6.31–8.3 % of *Cx. quinquefasciatus* populations would be expected to survive long enough for transmission to occur.**Conclusion**Our findings revealed that all the species tested were competent for RVFV with a significant more important role of *Ae. vexans* compared to *Cx. poicilipes *or* Cx. quinquefasciatus* and a highest potential of the East African lineage to be transmitted.

## Additional files

Additional file 1: Table S1. GenBank accession numbers of Rift Valley Fever Virus sequences used to design the primers used in this study. (DOC 19 kb) Additional file 2: Table S2. Mosquito tested, number infected, that disseminated and transmitted by species and Rift valley fever strains. (XLS 25 kb)
