# Supplementary material for: Vector competence of Aedes vexans (Meigen), Culex poicilipes (Theobald) and Cx. quinquefasciatus Say from Senegal for West and East African lineages of Rift Valley fever virus
Source: Parasit Vectors. 2016 Feb 20;9:94. doi: 10.1186/s13071-016-1383-y (PMC4761212; doi:10.1186/s13071-016-1383-y)
Supplement: Additional file 1: Table S1. — GenBank accession numbers of Rift Valley Fever Virus sequences used to design the primers used in this study. (DOC 19 kb) [file 13071_2016_1383_MOESM1_ESM.doc]

Table S1. GenBank accession numbers of Rift Valley Fever Virus sequences used to design the primers used in this study.

| Segment | GenBank accession numbers |
| --- | --- |
| S | EU574082.1, JF784388.1, EU574085.1, EU574070.1, EU312138.1, EU312136.1, EU312130.1, EU312127.1, EU312125.1, EU312121.1, EU312117.1, EU312115.1, EU312112.1, DQ380181.1, DQ380178.1, DQ380180.1 |
| M | DQ380202.1, DQ380205.1, JQ068143.1, EU574056.1, DQ380186.1, EU574056.1, EU574053.1, JF311385.1, JF311381.1, DQ380222.1, DQ380221.1, DQ380220.1, DQ380216.1, DQ380197.1, DQ380187.1, DQ380185.1 |
